# A case of secondary syphilis

**DOI:** 10.11604/pamj.2022.42.151.33017

**Published:** 2022-06-24

**Authors:** Krishna Prasanth Baalann, Mahalakshmi Krishnan

**Affiliations:** 1 Department of Community Medicine, Sree Balaji Medical College and Hospital, Bharath Institute of Higher Education and Research, Chennai, India,; 2 Department of Microbiology, Sree Balaji Dental College and Hospital, Bharath Institute of Higher Education and Research, Chennai, India

**Keywords:** Sexually transmitted diseases, secondary syphilis, macular rash

## Image in medicine

Syphilis is a chronic inflammatory disease caused by the organism *Treponema pallidum (T. pallidum*). Secondary syphilis is characterized by a rash that emerges around 3 to 6 weeks after the chancre forms, and may appear before the chancre heals in some cases. Other systemic symptoms may appear, indicating that the infection has migrated to other parts of the body. During the secondary stage, a person is extremely contagious. A 40-year-old man presented to our outpatient department with complaints of generalized weakness, headache, nausea, and arthralgia for the past few weeks. The patient had a history of having sex exclusively with men and had been in a monogamous relationship for the past 8 months. Physical examination revealed macular rash with scarred painless lesions over the glans penis and bilateral submandibular lymphadenopathy. Investigations revealed a positive reactive venereal disease research laboratory (VDRL) test. A fluorescent antibody test for *T. pallidum* showed positive for *T. pallidum* specific immunoglobulin G (IgG) antibody. HIV test was negative. For treatment, a single dose of 2.4 million units of Benzathine penicillin G was given intramuscularly and advised for follow-up. Patients with syphilis should have their sexual contacts assessed. Regardless of the results of serology testing, exposure within 90 days of a patient's diagnosis necessitates therapy.

**Figure 1:**
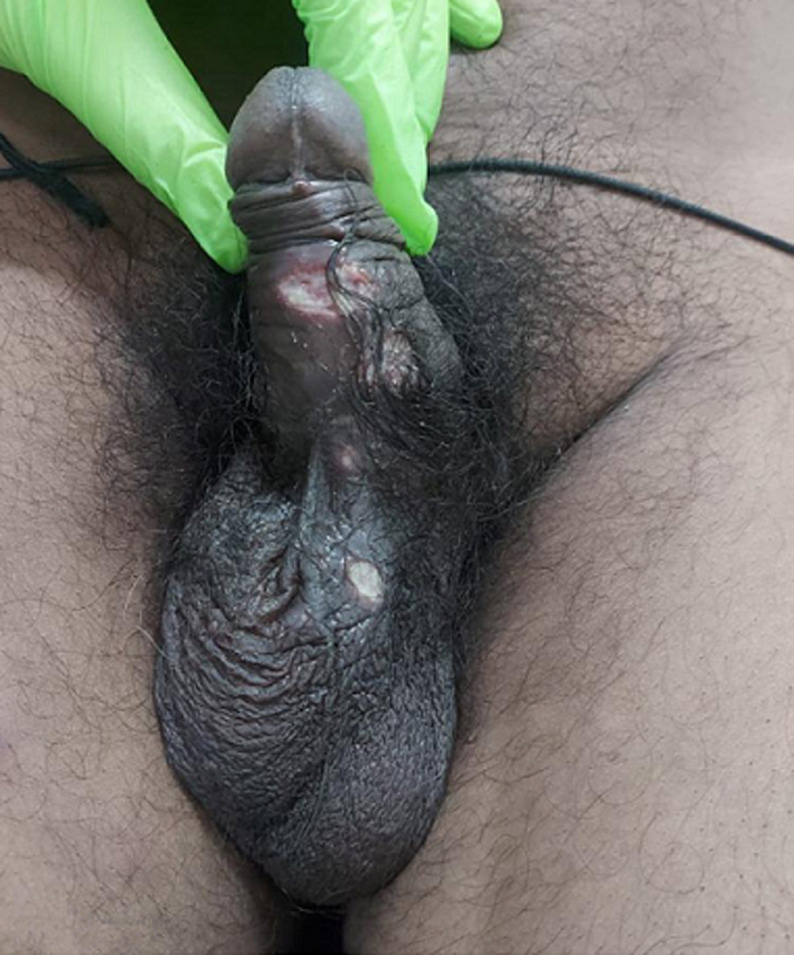
macular rash with scarred painless lesions over the glans penis

